# Characteristic of the Oxidative Stress in Blood of Patients in Dependence of Community-Acquired Pneumonia Severity

**DOI:** 10.3889/oamjms.2016.040

**Published:** 2016-03-11

**Authors:** Larissa Muravlyova, Vilen Molotov–Luchankiy, Ryszhan Bakirova, Dmitriy Klyuyev, Ludmila Demidchik, Valentina Lee

**Affiliations:** 1 *State Medical University, Biological Chemistry, Karaganda 100008, Kazakhstan*; 2 *State Medical University, Propaedeutics of Internal Diseases, Karaganda 100008, Kazakhstan*

**Keywords:** community-acquired pneumonia, modified proteins, malondialdehyde, blood plasma, erythrocytes

## Abstract

**BACKGROUND:**

At the present time the alternation of the oxidative metabolism is considered as one of the leading pathogenic mechanisms in the development and progression of community-acquired pneumonia (CAP). However the nature and direction of the oxidative protein changes in CAP patient’s blood had been almost unexplored.

**AIM:**

To define oxidative and modified proteins in erythrocytes and blood plasma of CAP patients.

**MATERIAL AND METHODS:**

Blood plasma and erythrocytes obtained from: 42 patients with moderate severity pneumonia, 12 patients with grave severity pneumonia and 32 healthy volunteers. Content of advanced oxidation protein products, malondialdehyde and reactive carbonyl derivatives were estimated as indicators of the oxidative stress and oxidative damage of proteins.

**RESULTS:**

In patients with grave severity the level of oxidative proteins and MDA in erythrocytes exceeded both: control values and similar meanings in CAP patients with moderate severity. The further growth of MDA in this group patients’ blood plasma was observed, but the level of oxidative proteins decreased in comparison with those in CAP patients with moderate severity.

**CONCLUSION:**

To sum up, our derived data show, that injury of erythrocytes’ redox-status and blood plasma components plays an essential role in development and progression CAP.

## Introduction

Nowadays the alteration of the oxidative metabolism (OM) is considered as one of the leading pathogenic mechanisms in the development and progression of community-acquired pneumonia (CAP). There were researches concerning the glutathione metabolism in CAP patients of young age, which helped to obtain the reliable data proved decreasing activity of glutathione in the blood against increasing activity of the superoxide dismutase and glutathione reductase. The same patients showed an increased index of peroxidation. Based on derived results the author concludes that leading factors in CAP belongs to disorder in immunity function and peroxide homeostasis [1]. The change in enzyme activity of antioxidant protection was found also in pilot research which had been made by Trefler S. et al. [2].

Reported that in CAP patients’ blood plasma and erythrocytes the content of malondialdehyde (MDA) and isoprostane has been decreased, as the ratio of oxidized and reduced glutathione has being changed, along with this the correlation between degree of changing and pneumonia severity has been found out. It is suggested to use the indicators of lipid peroxidation as biomarkers to assess the severity and clinical outcome in CAP [3, 4].

The children with CAP showed the malondialdehyde content expansion simultaneously with decreasing of antioxidant summary activity and the level of zinc. At the same time children with focal CAP had more expressed mentioned indicators, that allowed the authors to recommend detaching of these children in risk group for lingering illness [5].

There was an evaluation of the hydrogen peroxide and one of the lipid peroxidation indicators in expired air condensate of CAP patients. It was revealed that content of hydrogen peroxide in exhaled air was almost in 5 times higher than control values. Interestingly, on the 10^th^ day of treatment, this figure was higher than control one in 3.3 times. At the same moment the level of malondialdehyde was higher than control values on 1^st^ and 3^d^ day of treatment. Authors suggested that the main source of the hydrogen peroxide were activated leucocytes, monocytes and macrophages. The basic conclusion of this research was ascertaining of the oxidative stress development in lungs at CAP patients [6].

Analysis of literature data showed, that the nature and direction of the oxidative protein changes in CAP patient’s blood had been almost unexplored.

Protein oxidative modification – it is various kinds of post-translational modifications, which are caused by reaction of the amino acid residues with active oxygen and nitrogen forms. Moreover, protein oxidative modification might be determined by reaction with lipid peroxidation products, especially with reactive aldehyde [7]. The process of protein oxidative modification has biological significance, because, according to modern views, it is one of the most important mechanisms involved in cell signaling [8]. It is stated, that redox signaling is one of protection mechanisms against ischemic tissue lesion [9]. However, protein oxidative modification is often regarded as a negative component, since in conditions of oxidative stress the result is formation of large supramolecular complexes (aggregates), protein fragmentation etc. Eventually it causes the loss of functional protein activity that leads to severe metabolic disturbances [10].

The most stable products of the protein oxidative modification are reactive carbonyl derivatives. It should be noted, that carbonylation affects not only proteins, but also lipids and carbohydrates. The Semchyshyn H. M. [11] review described the currently known reactions of carbonyl derivatives formation in the organism, accumulation of which is positioned as a negative factor. There are new data about carbonyl derivatives (CD) ability to connect with a specific type of receptors, but this information needs to be verified. Advanced oxidation protein products (AOPP) present another type of oxidized protein. AOPP are formed from oxidative modification of albumin, but also the source might be fibrinogen or lipoproteins. It is suggested, that AOPP are formed with the participation of the myeloperoxidase and hypohaloids. AOPP structure is presented by dityrosine, carbonyl groups and cross-link. AOPP have quite distinct biological properties similar to advanced glycation end - products (AGEs) and can contact with AGE receptor (RAGE). It is believed, that the formation of AOPP might be increased in conditions of oxidative stress and inflammation [12]. AOPP have pro-oxidant and proinflammatory properties, as well as the ability to cause endothelial dysfunction [13, 14].

Membrane-bound hemoglobin is currently positioned as a variant of the modified protein. It is established, that membrane-bound hemoglobin is less protected by antiradical and antiperoxidant protection systems of red blood cells [15]. Hemoglobin is involved in the maintenance of red cells redox homeostasis. It is assumed, that hypoxia increases the proportion of membrane-bound hemoglobin, which in turn leads to erythrocyte membranes structural aberrations, redirection of calcium and potassium transportation, disruption of erythrocyte deformability [16].

Consequently, analysis of the literature data showed, that there are no researches about oxidative and modified proteins in erythrocytes and blood plasma of CAP patients, which was a reason enough for our investigation issue.

## Material and Methods

### Subjects

54 patients, at the age from 19 to 74 years old, with community-acquired pneumonia, treated in a specialized therapeutic and pulmonary department of city and regional hospitals of Karaganda have been studied. According to the design of study, the patients had been distributed in two groups: group (I) included the 42 patients with moderate serverity pneumonia and respiratory insufficiency grade (2), which was characterized by shortness of breath at rest, violation of restrictive capacity of lungs. The group (II) included 12 patients with severe pneumonia and respiratory insufficiency grade (2-3). Presence of infiltrative lung lesions, like tumors, diffuse connective tissue diseases, sarcoidosis, pulmonary tuberculosis, parasitic infestations and bronchiectasis were exclusion criterias of the study. Control group consisted of 32 healthy volunteers the same age as the group of CAP patients, who were without signs of any inflammation. All patients and healthy subjects have received the full information on probable inconveniences and complications at the blood sampling before giving their consent to participate.

42.6% patients with community-acquired pneumonia were smokers having smoker’s index more than 21 years. Half of these patients had smoking history more than 15 years. At the time of our study, more than 90% of patients stopped smoking for two days or more. More than 79% of patients were citizens of industrial towns, where they were living more than 10 years at the time of onset of disease.

The aspects of disease were presented not only by classic symptoms of coughing, fever ranging from high values to subfebrile fever (from 37° C to 40° C), shortness of breath but also by absence of symptoms of cough and respiratory failure. However, the first days of scant symptoms of pneumonia in some patients were limited to an expanded x-ray picture of inflammatory infiltration and changes in blood parameters. Furthermore, in a subsequent period spanning from 3 to 5 days after hospital admission of these patients, manifest clinical respiratory failure had been observed. The syndrome of cough in 14.8% of patients did not arise during the entire period of monitoring in hospital. In 75.9% of patients, location of pneumonic infiltrates at the basal segments of the right or left lung had been revealed, that allow diagnosing the 1-3 segmental pneumonia in the lower lobes. Affected region, which occupies the entire share or two shares, was detected in 22.2% of cases.

In 25.9% of the patients suffered from pneumonia, localization of disease had been revealed in the upper and/or secondary lobes. Bilateral pulmonary involvement, detected by X-ray, with the involvement of more than two segments occurs in 29.6% of patients. Thus, in our study, the pneumonia was a fairly extensive infiltrative lesion of the lung tissue. This provided significant intoxication syndrome, pyrexia and respiratory failure of patients.

The age of patients with bilateral pulmonary involvement was 49 ± 5.7 years old. Lower lobe pneumonia was typical for patients at the age from 31 to 72 years old. The sputum of all patients, without exception, had been examined for Mycobacterium tuberculosis.

Peculiarities of pneumonia in young people whose age does not exceed 32 years (n = 17) and averaged 28 ± 4.3 years were noticed. These patients had a prolonged clinical course and significant negative clinical and radiological improvement at the first week of inpatient stay in hospital. These patients did not belong to socially disadvantaged, did not have any previous alcohol incidents or habitual alcoholic intoxication. They never had cases of pneumonia or upper respiratory tract infections in their anamnesis. In the case of these patients, pneumonia started as a focal, then progressed in a short time and became a polysegmental or shared with the increase of intoxication and respiratory failure, which required the strengthening of antibiotic: prescription of the second antibiotic, increasing the dose and/or frequency of antibacterial substance injection.

The group of patients suffered from severe pneumonia (n = 12) involved persons at age from 21 to 63 years old, including, 4 men and 8 women. More than 75% of these patients were presented by young and middle age persons – from 27 to 42 years old. Area of pneumonia lesions covered one share and a number of other segments of the share. According to the distribution and localization it was bilateral pneumonia. Radiological improvement within two weeks after hospitalization was low, despite the reduction in symptoms of respiratory failure. For a long time it was mentioned the leukocytosis in the peripheral blood with significant prevalence of numbers of granulocytes in WBC differential and shifting of basilar cell, in spite of the significant positive improvement of ES (erythrocyte sedimentation) indicator.

### Radiological examination

Community-acquired pneumonia was diagnosed based on the presence of pulmonary infiltrates, radiologically verified. Conclusion of radiologist (survey radiography of the chest, lateral X-rays of the lungs, made with X-ray apparatus, equipped with a digital decoder, CT scan of the lungs) was a prerequisite for the diagnosis of pneumonia. Dynamical X-Ray control (digital X-ray photography, computed tomography) was performed in the period from 7 to 10 days.

### Blood sampling and Biochemical analysis

Blood was collected from the cubital vein (3 ml/sample) and was drawn into vacutainer tubes containing heparin. Erythrocytes were separated from plasma by centrifugation and washed for three times with physiological saline. Investigations of blood plasma and red blood cells were done within 1-2 hours after its collection.

AOPP were evaluated by Witko-Sarsat’s method [17]. The results were expressed in μmol/l. AOPP are formed from oxidative modification of albumin and/or fibrinogen. To define the primary type of proteins involved in AOPP forming, AOPP were measured both in blood plasma and in blood serum. MDA content in blood plasma was defined with colorimetric thiobarbituric acid method [18], units of measurement – nmol/ml.

Metabolic status of erythrocytes was assessed by the level of MDA – lipid peroxidation cytotoxic catabolite, which was evaluated using the colorimetric thiobarbituric acid method [19]. During calculation molar extinction coefficient (1.56 x 10^5^ M^-1^ cm^–1^) was used; units of measurement – μpmol/ml. The level of CD was measured by R.L. Levine’s protocol [20]. Measurements were performed using a UV -VIS Spectrophotometer Model PD-303UV.

Comparisons of the results obtained between patients and control participants were performed using non-parametric Mann-Whitney U-test (STATISTICA 7.0).

### Bacteriological examination

Bacteriological examination of sputum and/or bronchial washings showed that in 79.6% of patients, *Str. Pneumonia* e played etiologic role in the development of pneumonia, *E. coli*, *Klebsiella* were revealed in 13% of patients. Bacteriological examination of patients did not give a positive result, as at the stage of outpatient treatment, these patients received a series of broad-spectrum antibiotics: cephalosporin, fluoroquinolones and macrolides.

### Patients follow up

From the first hours of inpatient stay, immediately after the X-ray verification of the diagnosis of pneumonia, patients received empirical antibiotic therapy (protected aminopenicillins), which was subsequently adjusted based on the results of bacteriological research. At the same time, the patients received the substances for pulmonary surfactant reinforcement and for the purpose of sputum control. Detoxication therapy included intravenous drip infusions of mixtures of electrolyte solutions in combination with vitamins.

All patients had been recovered or dismissed from the hospital with stabilization of condition to aftercare and post-hospital rehabilitation under the medical control in out-patient and/or home conditions. Length of stay in hospital was ranged from 11 to 27 days.

## Results

It is evident from Table 1 data, that there is fixed reliable growth of CD in blood erythrocytes in proportion to CAP aggravation in 1.92 and 2.2 times respectively, in comparison with control.

**Table 1:** The indicators of oxidative stress in red blood cells of patients in dependence of CAP severity (X ± SD)

Groups	Carbonyl derivatives	Malondialdehyde	Membrane-bound hemoglobin
Control	8.24 ± 1.67	1.09 ± 0.005	12.63 ± 0.90

Patients with moderate severity of CAP	16.29 ± 1.31*	0.74 ± 0.06*	12.45 ± 1.59

Patients with severity CAP	17.97 ± 0.8*	1.82 ± 0.24*	14.34 ± 1.37

* - validity of differences with the control, p<0,05

At the same time in CAP patients with moderate severity the content of MDA was significantly decreased by 47% compared with control, whereas in CAP patients with grave severity this value, on the contrary, was considerably increased by 67%. There is a low tendency of membrane-bound hemoglobin content to enlarge in CAP patients’ erythrocytes with grave severity.

It is evident from Table 2 data, that the content of reactive CD was significantly increased in CAP patients’ blood plasma. In CAP patients with moderate severity the level of CD was significantly higher than control in 1.85 times, in CAP patients with grave severity – in 1.4 times.

**Table 2:** The indicators of oxidative stress in blood plasma of patients in dependence of CAP severity (X + SD)

Groups	Carbonyl derivatives	Advanced oxidation protein products	Malondialdehyde
Control	0.39 ± 0.08	0.21 ± 0.02	0.76 ± 0.10

Patients with moderate severity of CAP	0.72 ± 0.067*	0.8 ± 0.09*	0.91 ± 0.07*

Patients with severity CAP	0.55 ± 0.05*	0.5 ± 0.08*	1.12 ± 0.16*

* - validity of differences with the control, p < 0.05.

There was a reliable increasing of AOPP content in CAP patients’ blood plasma. Thus, in CAP patients with moderate severity AOPP level was higher than control in 3.8 times, in CAP patients with grave severity – in 2.38 times. Analysis of the AOPP content in the blood serum in both groups showed a similar meaning of this value that allowed suggesting the fibrinogen as the main source for AOPP.

The level of MDA was also considerably increased in the CAP patients’ blood plasma. Thus, in CAP patients with moderate severity MDA level was higher than control in 19.7%, in CAP patients with grave severity – in 47% in comparison with control values.

Analyzing the data, it is possible to make the following conclusion: there is a fixed elevation of the oxidative proteins in erythrocytes, simultaneously with increasing concentration of MDA in CAP patients with moderate severity. A similar trend of increased oxidative proteins and MDA was observed in CAP patients with grave severity.

In patients with grave severity the level of oxidative proteins and MDA in erythrocytes exceeded both: control values and similar meanings in CAP patients with moderate severity. There is a detected trend to membrane-bound hemoglobin extend in CAP patients’ erythrocytes with grave severity. The further growth of MDA in this group patients’ blood plasma was observed, but the level of oxidative proteins decreased in comparison with those in CAP patients with moderate severity.

## Discussion

Mainly CAP had pneumococcal etiology. It is established, that the hydrogen peroxide is used with pneumococcus as the virulent factor of epithelial cells lesion in the airways [21]. Generation of the hydrogen peroxide leads to the local oxidative stress development. The activation of neutrophils and other effector cells is also accompanied by local oxidative stress with excess generation of active oxygen forms. Active oxygen forms in the process of gas exchange migrate through the alveolar-capillary membrane and are able to induce the oxidative stress development in the erythrocytes [22].

The main reason for the intraerythrocytic oxidative stress development is oxidative destruction of the hemoglobin [23]. As the evidence of this thesis accuracy there is data about enlargement of CD content in erythrocytes. We suggest that more sensitive to the pro-oxidant action is not the cytosolic fraction, but membrane-bound hemoglobin one.

It is determined that after the damage of membrane-bound hemoglobin the hemochromes are formed and the process of heme degradation starts. Formation of hemochromes is accompanied by generation of superoxide anions and hydrogen peroxide; degradation of heme led to releasing of the most powerful pro-oxidant – free iron [16]. The result is activation of peroxidation (MDA accumulation), carbonyl stress development (increase of reactive CD), that was established by our researches.

In turn, the development of intracellular oxidative stress in erythrocytes leads to red blood cells’ membrane injury and releases the hemoglobin into blood plasma that causes redox reaction disruption. Another consequence of intracellular oxidative stress is the metabolic alternation and gas transport dysfunction of erythrocytes themselves [15]. On the one hand, it contributes to the hypoxemia development. On the other hand, diffusion of active oxygen forms from erythrocytes to endothelial cells of the lungs leads to additional attraction of leucocytes and contributes to the inflammatory process persistence. The principal possibility of such process is shown in the A. Huertas’study [24].

Accumulation of reactive CD, AOPP and MDA in patients’ blood plasma illustrates the oxidative stress transition from local to systemic pattern. As it was obtained in our researches, the main source of MDA production in CAP patients’ plasma is fibrinogen. It can be assumed that the blood plasma albumin is responsible for the reactive carbonyl derivatives formation. There are a number of adverse consequences due to increase of CD and AOPP in blood plasma, including further activation of neutrophils [25], which contributes to the inflammatory process persistence in CAP. Some decrease of CD and AOPP in CAP patients’ blood plasma, in our opinion, should be considered as an unfavorable sign, as it shows substrates insufficiency for their formation in conditions of oxidative stress intensification (progressive growth of MDA). The main source for reactive CD and AOPP formation are albumin and fibrinogen. The increase of oxidative albumin forms proportions testifies the decreasing antioxidant activity of blood plasma that contributes to high level of oxidative stress maintaining. The oxidative albumin is able to activate neutrophils. The predominance of oxidative fibrinogen form is a potential threat to hemostasis, including platelets [26] and erythrocytes aggregation.

To sum up, our derived data show, that injury of erythrocytes’ redox-status and blood plasma components plays an essential role in development and progression CAP mechanisms. It will very useful to estimate effectiveness of CAP treatment and possible diagnostics of CAP severity based on oxidative stress indicators.
